# Olaparib Addition to Maintenance Bevacizumab Therapy in Ovarian Carcinoma With *BRCA*-Like Genomic Aberrations

**DOI:** 10.1001/jamanetworkopen.2024.5552

**Published:** 2024-04-09

**Authors:** Philip C. Schouten, Sandra Schmidt, Kerstin Becker, Holger Thiele, Peter Nürnberg, Lisa Richters, Corinna Ernst, Isabelle Treilleux, Jacques Medioni, Florian Heitz, Carmela Pisano, Yolanda Garcia, Edgar Petru, Sakari Hietanen, Nicoletta Colombo, Ignace Vergote, Shoji Nagao, Sabine C. Linn, Eric Pujade-Lauraine, Isabelle Ray-Coquard, Philipp Harter, Eric Hahnen, Rita K. Schmutzler

**Affiliations:** 1 Department of Molecular Pathology, Netherlands Cancer Institute, Amsterdam, the Netherlands; 2 Center for Familial Breast and Ovarian Cancer, Center for Integrated Oncology, Medical Faculty, University Hospital Cologne, Cologne, Germany; 3 Cologne Center for Genomics, University of Cologne, Cologne, Germany; 4 Cologne Excellence Cluster on Cellular Stress Responses in Aging-Associated Diseases, University of Cologne, Cologne, Germany; 5 Center for Molecular Medicine Cologne, University Hospital, Cologne, Cologne, Germany; 6 Centre Léon BERARD, Lyon France; 7 Hôpital Européen Georges Pompidou, Paris and Groupe d'Investigateurs Nationaux pour les Etudes des Cancers de l'Ovaire, France; 8 Department of Gynecology & Gynecologic Oncology, EvangKliniken Essen-Mitte, Essen, Germany; 9 AGO Study Group, Wiesbaden, Germany; 10 Department of Urology and Gynecology, Istituto Nazionale Tumori IRCCS Fondazione G Pascale, Napoli, Italy; 11 Parc Taulí University Hospital, Sabadell, Spain and GEICO, Spain; 12 Department of Obstetrics and Gynecology, Medical University of Graz, Graz and AGO Austria, Austria; 13 Turku University Hospital, Turku, and Nordic Society of Gynaecological Oncology, Finland; 14 University of Milan-Bicocca and European Institute of Oncology Scientific Institute for Research, Hospitalization and Healthcare, Milan, and MaNGO, Italy; 15 University Hospital Leuven, Leuven Cancer Institute, Leuven, Belgium, European Union and BGOG, Belgium; 16 Department of Gynecologic Oncology, Hyogo Cancer Center, Hyogo, Japan a,d GOTIC, Japan; 17 Department of Medical Oncology, Netherlands Cancer Institute, Amsterdam, the Netherlands; 18 Department of Pathology, University Medical Center Utrecht, Utrecht, the Netherlands; 19 ARCAGY Research, Paris; 20 Centre Léon BERARD, and University Claude Bernard Lyon I, Lyon and GINECO, France

## Abstract

**Importance:**

Testing for homologous recombination deficiency is required for the optimal treatment of high-grade epithelial ovarian cancer. The search for accurate biomarkers is ongoing.

**Objective:**

To investigate whether progression-free survival (PFS) and overall survival (OS) of patients with high-grade epithelial ovarian cancer treated with maintenance olaparib or placebo differed between patients with a tumor *BRCA*-like genomic profile and patients without a tumor *BRCA*-like profile.

**Design, Setting, and Participants:**

This cohort study was a secondary analysis of the PAOLA-1 randomized clinical trial that compared olaparib plus bevacizumab with placebo plus bevacizumab as maintenance treatment in patients with advanced high-grade ovarian cancer after a good response to first-line platinum with taxane chemotherapy plus bevacizumab, irrespective of germline or tumor *BRCA1/2* mutation status. All patients with available tumor DNA were included in the analysis. The current analysis tested for an interaction between *BRCA*-like status and olaparib treatment on survival outcomes. The original trial was conducted between July 2015 and September 2017; at the time of data extraction for analysis in March 2022, a median follow-up of 54.1 months (IQR, 28.5-62.2 months) and a total follow-up time of 21 711 months was available, with 336 PFS and 245 OS events.

**Exposures:**

Tumor homologous recombination deficiency was assessed using the *BRCA*-like copy number aberration profile classifier. Myriad MyChoice CDx was previously measured. The trial was randomized between the olaparib and bevacizumab and placebo plus bevacizumab groups.

**Main Outcomes and Measures:**

This secondary analysis assessed hazard ratios (HRs) of olaparib vs placebo among biomarker strata and tested for interaction between *BRCA*-like status and olaparib treatment on PFS and OS, using Cox proportional hazards regression.

**Results:**

A total of 469 patients (median age, 60 [range 26-80] years) were included in this study. The patient cohort consisted of women with International Federation of Gynaecology and Obstetrics stage III (76%) high-grade serous (95%) ovarian cancer who had no evaluable disease or complete remission at initial or interval debulking surgery (76%). Thirty-one percent of the tumor samples (n = 138) harbored a pathogenic *BRCA* mutation, and *BRCA*-like classification was performed for 442 patients. Patients with a *BRCA*-like tumor had a longer PFS after olaparib treatment than after placebo (36.4 vs 18.6 months; HR, 0.49; 95% CI, 0.37-0.65; *P* < .001). No association of olaparib with PFS was found in patients with a non–*BRCA*-like tumor (17.6 vs 16.6 months; HR, 1.02; 95% CI, 0.68-1.51; *P* = .93). The interaction was significant (*P* = .004), and HRs and *P* values (for interaction) were similar in the relevant subgroups, OS, and multivariable analyses.

**Conclusions and Relevance:**

In this secondary analysis of the PAOLA-1 randomized clinical trial, patients with a *BRCA*-like tumor, but not those with a non–*BRCA*-like tumor, had a significantly longer survival after olaparib plus bevacizumab treatment than placebo plus bevacizumab treatment. Thus, the *BRCA1-*like classifier could be used as a biomarker for olaparib plus bevacizumab as a maintenance treatment.

## Key Points

**Question:**

Is olaparib maintenance treatment associated with longer survival in patients with ovarian cancer tumors with and without a previously established *BRCA*-like genomic profile?

**Findings:**

This cohort study was a secondary biomarker-by-treatment interaction analysis of 469 patients from a randomized clinical trial comparing the bevacizumab plus olaparib and bevacizumab plus placebo groups. Patients with *BRCA*-like tumors had a longer progression-free survival after olaparib vs placebo, but there was no significant difference in patients with a non–*BRCA*-like tumor after olaparib vs placebo.

**Meaning:**

The findings of this study suggest that the *BRCA*-like classifier can be used as a biomarker for olaparib maintenance therapy.

## Teaser

This cohort study examines overall and progression-free survival, using a *BRCA*-like genomic copy number aberration profile classifier, in patients with ovarian cancer receiving olaparib plus bevacizumab or placebo plus bevacizumab.

## Introduction

First-line maintenance therapy with a poly-ADP ribose polymerase (PARP) inhibitor and bevacizumab was approved for the treatment of advanced high-grade ovarian carcinoma (OC) by the US Food and Drug Administration and the European Medicines Agency following the results of the PAOLA-1 trial.^1^ Mechanistically, PARP inhibitors induce synthetic lethality in patients’ tumors harboring defects in homologous recombination, which are present in approximately 50% of high-grade serous OC.^2,3^ In the PAOLA-1 trial, improved progression-free survival (PFS) was observed when olaparib was added to maintenance bevacizumab in patients with homologous recombination–deficient (HRD) tumors, with or without tumor *BRCA1/2* mutations based on Myriad MyChoice CDx, but not in patients with homologous recombination–proficient (HRP) tumors.^4^ A wide range of potential tests to identify HRD tumors are available, ranging from mutational testing and mutational and copy number signatures to functional assays.^5^ However, there is a need for improvement of defining the patient subgroups, as globally there is a lack of consistently defined subgroups, a lack of negative predictive value in most of these tests, and an inadequate assessment of the complex and dynamic nature of HRD phenotype.^5^ Despite these shortcomings, the European Medicines Agency stated that HRD should be determined with a validated test by an experienced laboratory, without specifying a test.^6^ Decentralized testing, in which an assay can be controlled within its own laboratory environment, is an important benefit for research groups and molecular pathologists. Furthermore, health care systems do not always allow reimbursement for tests conducted in central laboratories abroad, such as the Myriad MyChoice CDx test, which is performed in the US exclusively.^7^ Therefore, the European Network for Gynaecological Oncological Trial (ENGOT) Group HRD Initiative aims to identify new, reliable, and feasible HRD tests developed by European academic laboratories associated with the ENGOT trial groups.^8^

We developed a *BRCA*-like genomic copy number aberration profile classifier (*BRCA*-like classifier) that classifies whether a tumor has aberrations similar to those observed in tumors from patients with *BRCA1* and *BRCA2* germline pathogenic variants.^9^ The signature is assumed to identify abnormalities like those found in *BRCA1/2*-mutated tumors and may identify tumors that are HRD. When applied to the observational AGO-TR1 study, the classifier assigned *BRCA*-like status to tumors with germline and somatic *BRCA1* and *BRCA2* mutations, *BRCA1* promoter hypermethylation, and pathogenic germline variants in *RAD51C*.^9^ When the test was applied to patients in the phase 3 OVHIPEC trial (NCT00426257), which randomized the addition of heated intraperitoneal chemotherapy (HIPEC) to standard neoadjuvant carboplatin with paclitaxel or standard neoadjuvant carboplatin with paclitaxel treatment, patients with non–*BRCA*-mutant *BRCA*-like tumors had longer PFS after the addition of HIPEC treatment.^10^ This serves as preliminary evidence that this test may be a predictive biomarker for HRD-targeting therapy.^10^ However, the use of HIPEC therapy in general has been debated extensively^11^ and, unexpectedly, did not have longer survival in patients with germline pathogenic variants in *BRCA1/2*, likely due to a prognostic benefit that is, as of yet, of uncertain origin.^10^ In the current investigation, we applied our test to a subgroup of 469 patients enrolled in the PAOLA-1 trial to investigate whether the addition of olaparib maintenance therapy in first-line advanced OC is associated with longer survival in patients with *BRCA*-like tumors than in patients with non–*BRCA-*like tumors.

## Methods

### Setup of the ENGOT European HRD Initiative

The ENGOT European HRD Initiative is a prespecified exploratory analysis of the randomized clinical PAOLA-1 study (NCT02477644) and consists of 3 phases.^8^ In the first phase, putative tests beyond HRD gene panel sequencing were identified and selected to proceed to phase 2. In phase 2, DNA samples from 85 tumors were available to assess concordance with Myriad MyChoice CDx, to determine whether the test was associated with PFS after olaparib treatment, and to train and improve the test, if necessary. To proceed to phase 3 of the HRD Initiative, tests required either high concordance with Myriad MyChoice CDx as assessed by Cohen κ testing and/or association with PFS after olaparib treatment for patients with biomarker-positive status and no association with longer PFS for those who were biomarker-negative. These criteria were defined upfront to allow for the selection of tests that were trained on the labels assigned by Myriad MyChoice CDx or achieved very similar performance, but also to allow tests that have a predictive biomarker-style survival association but do not necessarily behave similarly to Myriad MyChoice CDx. This was required because the HRD label comprises both a measurable analytical validity component (eg, mutational status of *BRCA1/2*), but also a functional component of association with survival on specific treatment. By aiming to just reproduce the Myriad MyChoice CDx class label, one would not be able to identify a test with a different or better split in survival outcome after olaparib between patients with biomarker-positive and biomarker-negative status. The κ value and associations with survival were assessed by Arcagy, independently of the authors of the *BRCA*-like classifier. Subsequently, 384 additional tumor DNA samples were analyzed in phase 3 of the program. Prespecified analyses were associations with survival after olaparib treatment in patients with biomarker-positive and -negative status, and a comparison with Myriad MyChoice CDx was performed to investigate relevant differences. The trial was performed in accordance with the provisions of the Declaration of Helsinki^12^ and Good Clinical Practice guidelines under the auspices of an independent data monitoring committee. Written informed consent was obtained from all patients. The ethics committee Comite de Protection des Personnes SUD-EST IV gave ethical approval for this work. This study followed the Strengthening the Reporting of Observational Studies in Epidemiology (STROBE) reporting guideline.

### Samples and Clinical Data

Between July 2015 and September 2017, the PAOLA-1 double-blind placebo-controlled trial randomized patients with newly diagnosed advanced (International Federation of Gynaecology and Obstetrics [FIGO] stage III or IV) high-grade serous or endometrioid OC, primary peritoneal carcinoma, or fallopian tube carcinoma.^13^ Briefly, stratified block randomization was performed according to the outcome of first-line treatment and tumor *BRCA* status in a 2:1 ratio to receive olaparib, 300 mg twice daily, or placebo. The primary end point was PFS, which was the time of randomization until disease progression or death was assessed. Overall survival (OS) was the secondary end point. For censoring rules, refer to eTable 2 in Supplement 1. Survival data were updated in March 2022. All patients with sufficient tumor DNA samples were included in the present study. We received 100 ng of DNA and the clinical data comprising mutation status, response to first-line treatment, age, performance status, timing of surgery, result of surgery, FIGO stage, histologic subtype, Myriad MyChoice CDx HRD test result, pretreatment CA-125 measurement, PFS, and OS.

### Quality Control and *BRCA*-Like Classification

Tumor DNA samples were sequenced as described before^9^ (eMethods, eFigures 1-5, and eTable 1 in Supplement 1). Copy number aberration profiles were generated by aligning low-coverage whole-genome sequencing reads to the hg38 reference genome, read counting in bins, normalization of mappability and guanine-cytosine content within the data set, and subsequent normalization to the reference training data set. This input was used for the shrunken centroid classifier that classified the profile as *BRCA*-like (posterior probability >0.5) or non–*BRCA*-like (posterior probability ≤0.5) as described before.^9^ Quality control was performed using noise variance and signal-to-noise ratio of the obtained copy number profiles in conjunction with visual assessment.

### Statistical Analysis

Baseline characteristics were tabulated and assessed using Fisher exact test, χ^2^ test, and continuous variables with Wilcoxon rank sum tests after examining the deviation from the normality assumption using the Shapiro-Wilk test. The Kaplan-Meier method was used to assess PFS and OS. The log-rank test was used to assess differences in survival between the olaparib and placebo groups. We used Cox proportional hazards regression analysis to estimate the hazard ratio (HR), 95% CI, *P* value, and *P* value for interaction (cross-product term) between the olaparib and placebo groups. In multivariable analyses, we adjusted for FIGO stage, CA-125 measurements at baseline, and response to first-line therapy. Analyses were performed in *BRCA-*like and non–*BRCA*-like groups. This study was an intention-to-treat analysis. All tests were 2-sided, with *P* values less than .05 considered significant. Schoenfeld residuals were used to assess the proportional hazards assumption, with global or individual *P* < .05. We used dfbeta residual analysis to assess outliers. Analyses were performed in R, version 4.2.1 (R Foundation for Statistical Computing).^14^

## Results

A total of 469 of the 806 randomized patients were available for this study. A flowchart of the study cohort is shown in eFigure 6 in Supplement 1. The patient cohort consisted of women with FIGO stage III (76%) high-grade serous (95%) OC who had no evaluable disease or complete remission at initial or interval debulking surgery (76%). The median age of the cohort was 60 (range, 26-80) years. Thirty-one percent of the tumor samples (n = 138) harbored a pathogenic *BRCA* mutation. *BRCA*-like classification was performed for 442 patients. A median follow-up of 54.1 months (IQR, 28.5-62.2 months), and a total follow-up time of 21 711 months was available, with 336 PFS and 245 OS events. Six patients were lost to follow-up, 6 patients withdrew informed consent in the olaparib arm, and 3 patients withdrew consent in the placebo arm.

We pooled samples from phases 2 and 3 of the ENGOT HRD project (n = 85 and n = 384, total n = 469) because we did not optimize the classifier during phase 2. Eight samples failed sequencing. An additional 19 samples were excluded by combined statistical and visual quality control of the copy number aberration profile. Noisy profiles were obtained for 3 of these. For the remaining 16, the resulting profiles showed few or low-level aberrations, indicating low or lower than the estimated tumor cell content.

Table 1 describes the overall cohort characteristics and the cohort without *BRCA-*mutated tumors, showing balanced subgroups for histologic status, FIGO stage, and first-line therapy response. Pathogenic variants in *BRCA1* and *BRCA2* were enriched in patients with *BRCA-*like tumors (129 *BRCA*-like vs 9 non–*BRCA*-like; *P* < .001) and patients with *BRCA*-like tumors were significantly younger than those with non–*BRCA*-like tumors in the full cohort (58.7 vs 62.1 years; *P* < .001). All other clinicopathologic characteristics were evenly distributed among patients with *BRCA-*like and non–*BRCA-*like tumors.

**Table 1.** Patient Characteristics (N=469) of the Full Cohort and Cohort Without *BRCA-*Mutated Tumors

Characteristic	Full cohort			Subgroup without *BRCA-*mutated tumors								
				*BRCA*-like and Myriad MyChoice available			Discordant samples			Concordant samples		
	No. (%)		*P* value	No. (%)		*P* value	No. (%)		*P* value	No. (%)		*P* value
	Not *BRCA*-like	*BRCA*-like		Not *BRCA*-like	*BRCA*-like		Not *BRCA*-like; Myriad HRD	*BRCA*-like; Myriad not HRD		Not *BRCA*-like; Myriad not HRD	*BRCA*-like; Myriad HRD	
No. (%)	144 (33)	298 (67)	NA	121 (45)	150 (55)	NA	14 (17)	68 (83)	NA	107 (57)	82 (43)	NA
Age, median (range), y	62.1 (26-78)	58.7 (32-80)	<.001	62.3 (26-78)	6.6 (32-78)	.07	62.5 (51-73)	64.5 (45-78)	.96	64.0 (26-78)	59.0 (32-77)	.006
High-grade serous	138 (96)	283 (95)	.73	116 (96)	143 (95)	.54	14 (100)	66 (98)	>.99	102 (95)	77 (94)	.48
High-grade endometrioid	3 (2)	10 (3)		2 (2)	5 (3)		0	1 (1)		2 (2)	4 (5)	
Other	3 (2)	5 (2)		3 (2)	2 (2)		0	1 (1)		3 (3)	1 (10)	
FIGO stage												
III	111 (77)	224 (75)	.72	94 (78)	112 (75)	.67	12 (86)	50 (74)	.50	82 (77)	62 (76)	.87
IV	33 (23)	74 (25)		27 (22)	38(25)		2 (14)	18 (26)		25 (23)	20 (24)	
First-line response												
NED with complete macroscopic resection at initial debulking surgery	58 (40)	115 (39)	.88	53 (44)	55 (37)	.59	7 (50)	17 (25)	.23	46 (43)	38 (46)	.77
NED/CR with complete macroscopic resection at interval debulking surgery	26 (18)	62 (21)		16 (13)	24 (16)		2 (14)	11 (16)		14 (13)	13 (16)	
NED/CR in patients with incomplete resection or no debulking surgery	23 (16)	52 (17)		19 (16)	30 (20)		2 (14)	17 (25)		17 (16)	13 (16)	
Partial response	35 (24)	67 (22)		31 (25)	40 (26)		2 (14)	23 (34)		29 (27)	17 (21)	
Missing	2 (1)	2 (0)		2 (2)	1 (1)		1 (8)	0		1 (1)	1 (1)	
CA-125 level at baseline												
Normal (≤ULN)	130 (90)	263 (88)	.89	109 (90)	130 (87)	.45	11 (78)	55 (81)	>.99	98 (92)	75 (91)	>.99
Abnormal (>ULN)	14 (10)	34 (11)		12 (10)	20 (13)		3 (22)	13 (19)		9 (8)	7 (9)	
Missing	NA	1 (1)		NA	NA		NA	NA		NA	NA	
*BRCA1/2* mutation status												
Not pathogenic	135 (94)	169 (57)	<.001	NA	NA	NA	NA	NA	NA	NA	NA	NA
Pathogenic	9 (6)	129 (43)		NA	NA	NA	NA	NA	NA	NA	NA	NA

Abbreviations: CR, complete remission; FIGO, International Federation of Gynaecology and Obstetrics; HRD, homologous recombination–deficient; NA, not applicable; NED, no evaluable disease; ULN, upper limit of normal.

**Figure 1.**
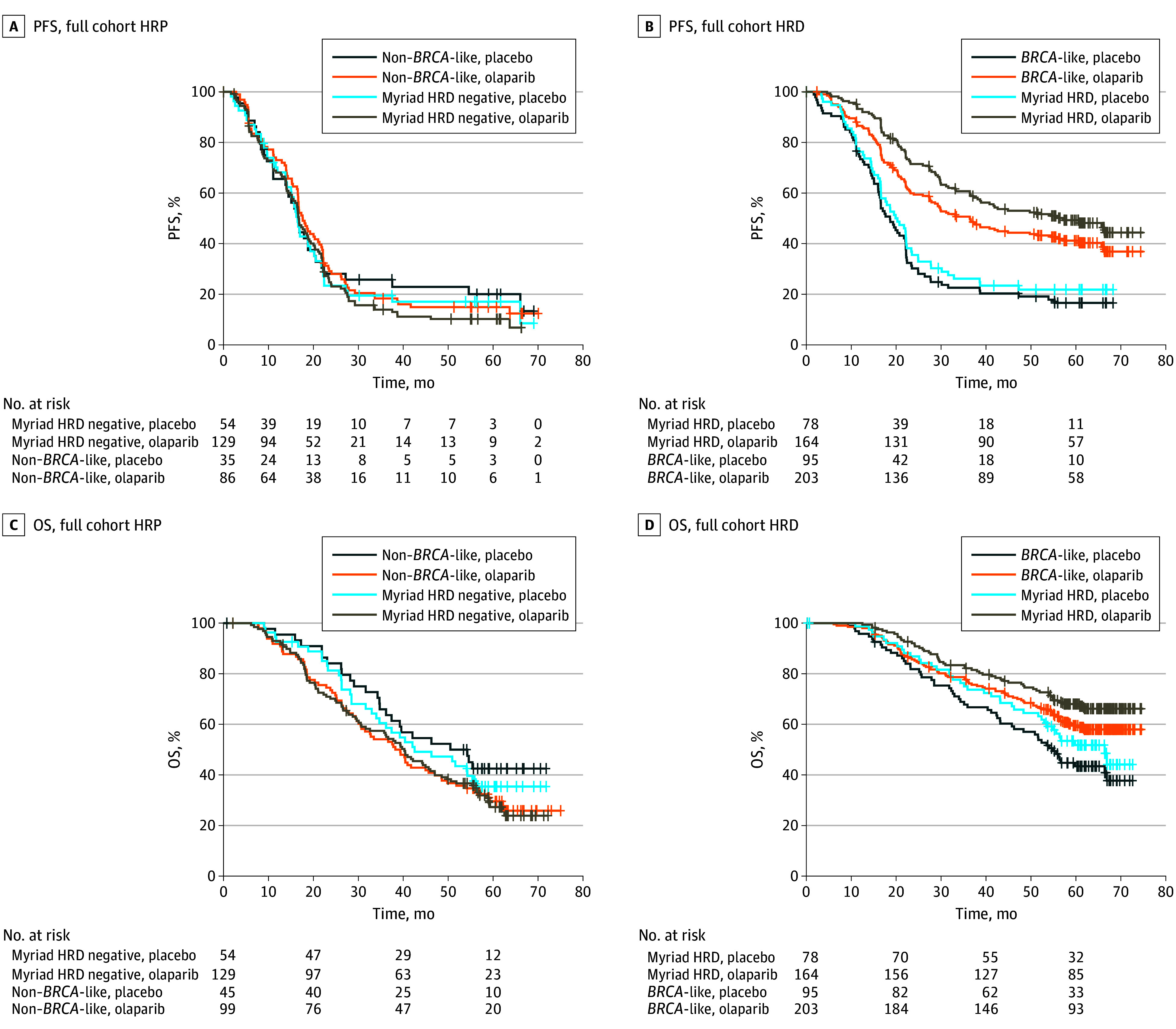
Progression-Free Survival (PFS) and Overall Survival (OS) of the Full Cohort Progression-free survival of patients with homologous recombination–proficient (HRP) tumors (A) and patients with homologous recombination–deficient (HRD) (B) tumors treated with olaparib plus bevacizumab or placebo plus bevacizumab. Overall survival of patients with HRP tumors (C) and patients with HRD tumors (D) treated with olaparib plus bevacizumab or placebo plus bevacizumab. Myriad indicates Myriad MyChoice CDx.

Figure 1 shows the Kaplan-Meier analysis of PFS and OS after olaparib plus bevacizumab and bevacizumab plus placebo for the *BRCA*-like classifier and Myriad MyChoice test in all available patients. Table 2 reports the median survival, number of events, and univariable and multivariable HRs within the biomarker treatment subgroups and the *P* values for interaction.

**Table 2.** Cox Proportional Hazards Regression Analysis of Full Cohort and Cohort Without *BRCA-*Mutated Tumors

Variable	Median (IQR), mo	No. of events/No. of patients (person-months)	Univariable		*P* value for interaction^a^	Multivariable^b^		*P* value for interaction^a^
			HR (95% CI)	*P* value		HR (95% CI)	*P* value	
**Full cohort**								
Progression-free survival								
Non–*BRCA*-like, placebo	16.6 (9.0-37.5)	35/45 (1028)	1 [Reference]	.93	.004	1 [Reference]	.76	.003
Non–*BRCA*-like, olaparib	17.6 (11.2-27.0)	82/99 (2194)	1.02 (0.68-1.51)			0.94 (0.63-1.41)		
*BRCA*-like, placebo	18.6 (11.8-27.7)	77/95 (2282)	1 [Reference]	<.001		1 [Reference]	<.001	
*BRCA*-like, olaparib	36.4 (16.7-75.2)	119/203 (7403)	0.49 (0.37-0.65)			0.44 (0.33-0.59)		
Myriad not HRD, placebo	16.5 (9.7-22.4)	44/54 (1991)	1 [Reference]	.66	<.001	1 [Reference]	.99	<.001
Myriad not HRD, olaparib	16.8 (8.9-23.5)	112/129 (3882)	1.08 (0.76-1.53)			1 (0.70-1.44)		
Myriad HRD, placebo	20.1 (12.8-38.6)	57/78 (2020)	1 [Reference]	<.001		1 [Reference]	<.001	
Myriad HRD, olaparib	57.1 (22.1-75.2)	84/164 (6867)	0.42 (0.3-0.59)			0.41 (0.29-0.58)		
Overall survival								
Non-*BRCA*-like, placebo	52.3 (30.3-NR)	25/45 (1991)	1 [Reference]	.09	.004	1 [Reference]	.12	.003
Non-*BRCA*-like, olaparib	38.8 (22.9-NR)	70/99 (3882)	1.48 (0.94-2.33)			1.45 (0.91-2.30)		
*BRCA*-like, placebo	54.9 (31.8-NR)	54/95 (4394)	1 [Reference]	.01		1 [Reference]	.003	
*BRCA*-like, olaparib	75.2 (39.3-75.2)	82/203 (7403)	0.64 (0.45-0.90)			0.59 (0.42-0.84)		
Myriad not HRD, placebo	42.5 (26.3-NR)	34/54 (2272)	1 [Reference]	.28	.01	1 [Reference]	.46	.04
Myriad not HRD, olaparib	40.2 (20.5-62.5)	91/129 (2536)	1.25 (0.84-1.85)			1.16 (0.78-1.74)		
Myriad HRD, placebo	66.4 (35.5-NR)	38/78 (2020)	1 [Reference]	.02		1 [Reference]	.03	
Myriad HRD, olaparib	75.2 (48.6-NR)	54/164 (8833)	0.6 (0.39-0.91)			0.62 (0.41-0.95)		
**Subgroup without *BRCA-*mutated tumors**								
Progression-free survival								
Non-*BRCA*-like, placebo	16.6 (8.7-27.2)	32/40 (787)	1 [Reference]	.89	.02	1 [Reference]	.93	.003
Non-*BRCA*-like, olaparib	17.6 (11.1-26.1)	81/95 (1820)	1.03 (0.68-1.55)			0.98 (0.642-1.50)		
*BRCA*-like, placebo	16.2 (10.2-23.4)	54/59 (986)	1 [Reference]	<.001		1 [Reference]	<.001	
*BRCA*-like, olaparib	21.2 (14.0-NR)	76/110 (2875)	0.53 (0.37-0.76)			0.43 (0.30-0.62)		
Myriad not HRD, placebo	16.2 (9.7-22.2)	43/151 (1007)	1 [Reference]	.88	<.001	1 [Reference]	.74	.001
Myriad not HRD, olaparib	16.8 (9.1-23.5)	107/124 (4814)	0.97 (0.68-1.39)			0.94 (0.65-1.37)		
Myriad HRD, placebo	16.6 (10.9-27.7)	32/36 (766)	1 [Reference]	.001		1 [Reference]	<.001	
Myriad HRD, olaparib	38.9 (16.7-NR)	34/60 (3048)	0.39 (0.24-0.63)			0.33 (0.20-0.54)		
Overall survival								
Non-*BRCA*-like, placebo	48.4 (25.2-NR)	23/40 (1582)	1 [Reference]	.09	.03	1 [Reference]	.09	.008
Non-*BRCA*-like, olaparib	38.0 (27.4-NR)	69/95 (3391)	1.51 (0.94-2.42)			1.52 (0.94-2.45)		
*BRCA*-like, placebo	48.4 (30.0-NR)	38/59 (2188)	1 [Reference]	.17		1 [Reference]	.03	
*BRCA*-like, olaparib	56.4 (21.1-62.5)	58/110 (4471)	0.75 (0.49-1.13)			0.64 (0.42-0.97)		
Myriad not HRD, placebo	42.5 (26.3-NR)	33/51 (2133)	1 [Reference]	.33	.08	1 [Reference]	.44	.06
Myriad not HRD, olaparib	40.2 (20.3-62.5)	89/124 (4814)	1.22 (0.82-1.82)			1.18 (0.78-1.78)		
Myriad HRD, placebo	53.5 (35.2-NR)	20/36 (1636)	1 [Reference]	.14		1 [Reference]	.07	
Myriad HRD, olaparib	NR (35.2-NR)	24/60 (3048)	0.64 (0.35-1.16)			0.57 (0.31-1.05)		

Abbreviations: HR, hazard ratio; HRD, homologous recombination–deficient; NR, not reached.

^a^ *P* value for interaction between *BRCA*-like and treatment arm; the placebo arm is the reference (HR = 1).

^b^ Multivariable analysis is adjusted for International Federation of Gynaecology and Obstetrics stage, CA-125 level at baseline and response at first-line treatment.

Patients with *BRCA*-like tumors had longer PFS after olaparib than after placebo treatment (median, 36.4 vs 18.6 months; HR, 0.49; 95% CI, 0.37-0.65; *P* < .001), whereas patients with a non–*BRCA*-like tumor had no significant difference in PFS after treatment with olaparib and placebo (median, 17.6 vs 16.6 months; HR, 1.02; 95% CI, 0.68-1.51; *P* = .93). In patients with non–*BRCA*-like tumors, a median OS of 38.8 months was observed after treatment with olaparib vs 52.3 months after placebo (HR, 1.48; 95% CI, 0.94-2.33; *P* = .09). For patients with *BRCA*-like tumors, the median OS was 75.2 vs 54.9 months (HR, 0.64; 95% CI, 0.45-0.90; *P* = .01). The interaction between *BRCA*-like status and treatment was significant at *P* = .004 for OS and *P* = .004 for PFS. No significant difference in prognosis was observed for *BRCA*-like tumor status alone. Hazard ratios and *P* values for interaction were similar in multivariable analyses (Table 2).

**Figure 2.**
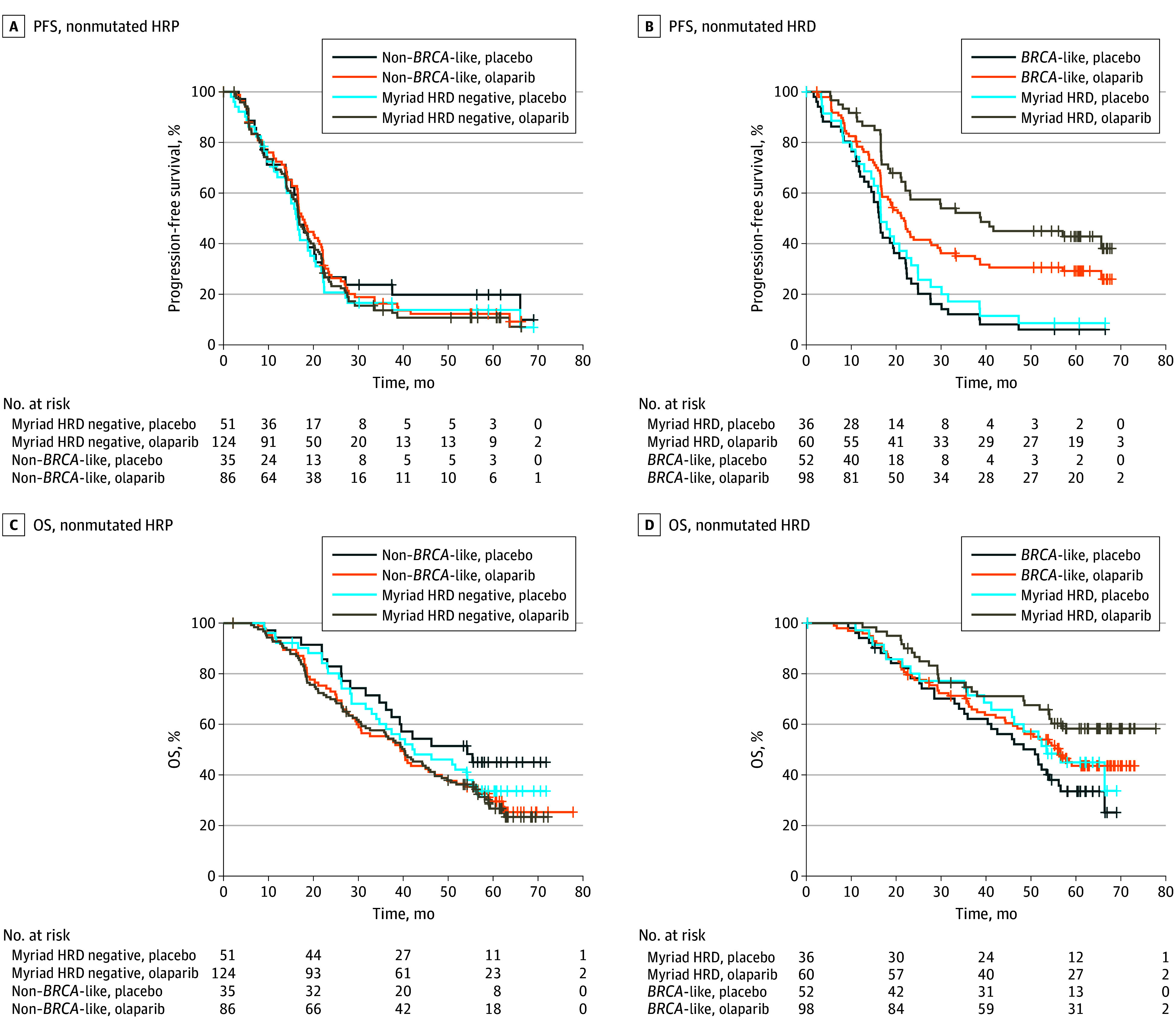
Progression-Free Survival (PFS) and Overall Survival (OS) of the Full Cohort and the Cohort Without *BRCA-*Mutated Tumors Progression-free survival (PFS) of patients with homologous recombination–proficient tumors (HRP) (A) and patients with homologous recombination–deficient (HRD) (B) tumors treated with olaparib plus bevacizumab or placebo plus bevacizumab. Overall survival of patients with HRP tumors (C) and patients with HRD tumors (D) treated with olaparib plus bevacizumab or placebo plus bevacizumab. Myriad indicates Myriad MyChoice CDx.

Although the *BRCA*-like classifier was trained to be an independent test for HRD status, the likely clinical use will be to identify non–*BRCA1/2*-mutated HRD tumors, either with parallel tumor/germline DNA for *BRCA1/2* mutation status for a comprehensive view or a stepwise testing algorithm if no *BRCA1/2* mutation is identified in resource-limited situations. Therefore, we investigated the subgroup without *BRCA-*mutated tumors. Figure 2 shows the Kaplan-Meier curves for PFS and OS of patients without *BRCA1/2* mutations. Patients with non–*BRCA*-like tumors had a median PFS of 16.6 months after receiving placebo and of 17.6 months after olaparib (HR, 1.03; 95% CI, 0.68-1.55; *P* = .89). In patients with a *BRCA*-like tumor, we observed a median PFS of 16.2 months after placebo and 21.2 months after olaparib (HR, 0.53; 95% CI, 0.37-0.76; *P* < .001) (*P* = .02 for interaction). For OS, the HR for non–*BRCA*-like tumors was 1.51 between olaparib and placebo treatment (95% CI, 0.94-2.42; *P* = .09) and 0.75 for *BRCA*-like tumors (95% CI, 0.49-1.13; *P* = .17). When adjusted in multivariable analysis, the HR for non–*BRCA*-like tumors remained similar (HR, 1.52; 95% CI, 0.94-2.45; *P* = .09), whereas the HR for *BRCA*-like tumors reached significance (HR, 0.64; 95% CI, 0.42-0.97; *P* = .03) (*P* = .008 for interaction).

### Comparison With Myriad MyChoice CDx

**Figure 3.**
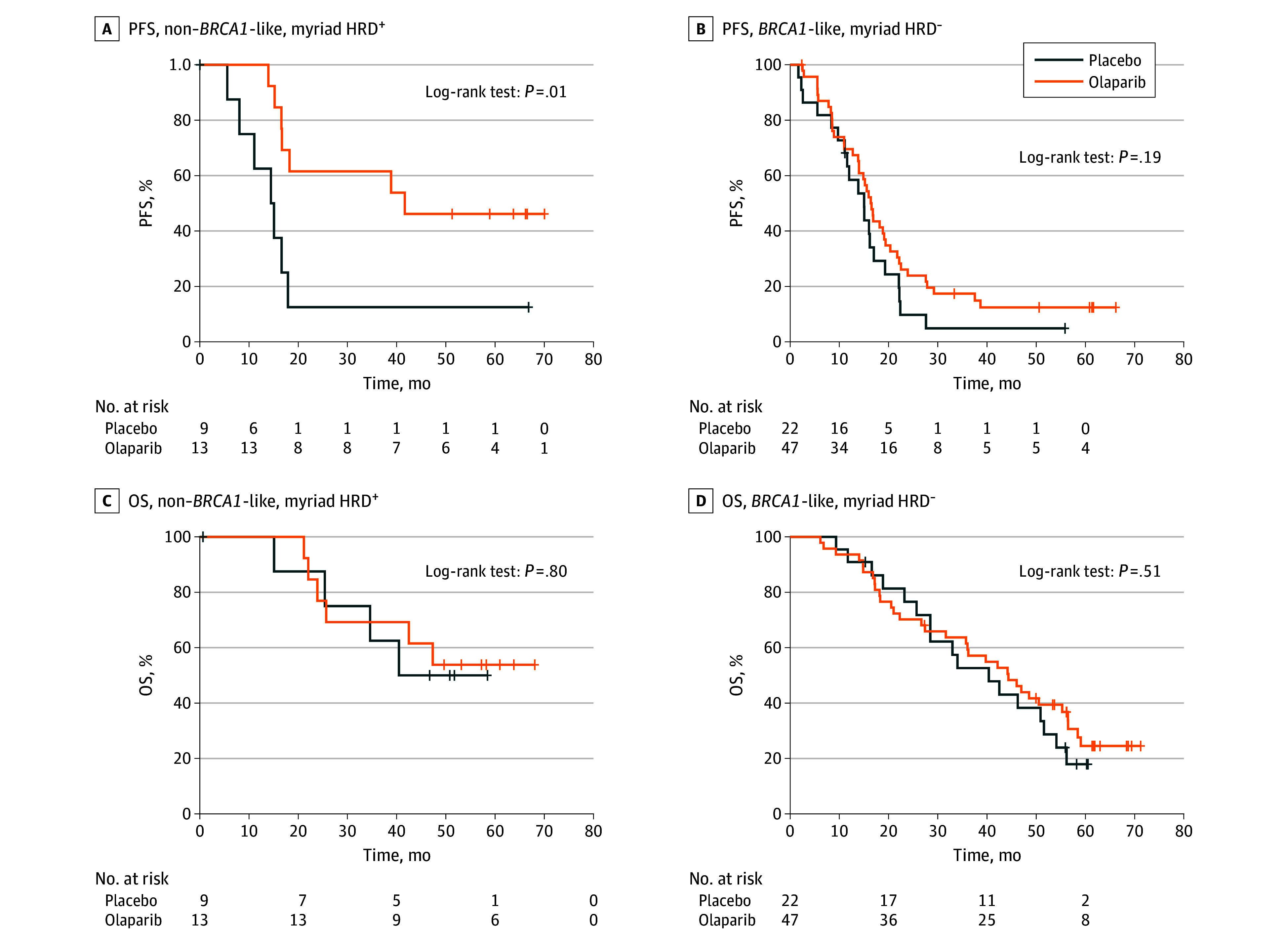
Progression-Free Survival (PFS) and Overall Survival (OS) in Patients With a Discordant Conclusion Between the 2 Tests Progression-free survival (A and B) and OS (C and D) of patients with discordant conclusions between the 2 tests.

The *BRCA-*like classifier analysis was unsuccessful in 27 of 469 cases (5.7%) and the MyChoice test was unsuccessful in 44 of 469 cases (9.4%). Both assays were unsuccessful in only 7 patients. Of the 405 samples that were successfully analyzed using both assays, concordant results were obtained for 107 HRD *BRCA*-like negative and 207 HRD *BRCA*-like positive (314 of 405 [77.5%]; 95% CI, 73%-81%) tumors. Of the remaining patients, 22 had Myriad MyChoice HRD and non–*BRCA-*like tumors, and 69 tumors were classified as *BRCA-*like Myriad MyChoice HRP (eTable 3 in Supplement 1). Although the *BRCA*-like classifier identified more patients with HRD, both assays showed associations with survival after olaparib and placebo, specifically in the multivariable-adjusted model, as shown in Figures 1 and 2 and Table 2. In the nonmutated samples that were non–*BRCA*-like Myriad MyChoice HRD, we observed an HR of 0.19 for PFS (95% CI, 0.06-0.65; *P* = .008) comparing olaparib and placebo treatment. Samples with a *BRCA*-like Myriad MyChoice HRP equally had a significantly different HR after olaparib compared with placebo, although the effect size was more modest (HR, 0.56; 95% CI, 0.32-0.97; *P* = .04) (Figure 3; eTable 4 in Supplement 1).

## Discussion

In this retrospective analysis of a randomized clinical trial comparing olaparib plus bevacizumab with placebo plus bevacizumab, we observed that the addition of olaparib was associated with longer OS and PFS in patients with *BRCA*-like tumors, but not in non–*BRCA*-like tumors. We did not perform training or optimization of the test on the PAOLA-1 cohort and therefore prevented bias in the outcomes of the test toward the characteristics of the patients included in this trial.

Unexpectedly, we found that patients with non–*BRCA*-like tumors had a higher event rate for OS after receiving olaparib than after receiving placebo, which, although not significant, would be clinically relevant if further substantiated in follow-up studies. Mechanistically, this may be explained by the selection of resistance mechanisms after applying a cytotoxic agent (PARP inhibition) in patients without relevant sensitive background.^15^

We compared our test with the approved biomarker and found similar associations with survival after olaparib in patients with biomarker-positive status, with overlapping CIs. Differences were a lower dropout rate and a larger biomarker-positive group, while retaining associations with longer survival after olaparib treatment in the biomarker-positive group, and a lack of difference in survival in the biomarker-negative group. Furthermore, the *BRCA*-like classifier may identify a group of patients with poor OS after olaparib treatment if substantiated in further studies. In addition, we observed that the assay could be performed in a decentralized manner, facilitating its implementation in current workflows.

### Strengths and Limitations

The PAOLA-1 phase 3 trial offered a unique opportunity to further investigate and validate new academically developed biomarkers for HRD at the European level.^8,16,17,18^ The study design of the HRD Initiative and the randomized nature of the trial generated the highest level of evidence possible outside of a prospective trial for those biomarkers that showed predictive biomarker characteristics.^19,20^

However, this study has limitations. This is a post hoc exploratory analysis that, although prespecified, should be carefully interpreted regarding statistical testing and multiplicity (subgroups and assessing other biomarkers in the same trial). Furthermore, only a subgroup of patients was available. Within this subgroup, we could not obtain molecular results for all patients. Although the measured samples were representative of the full trial population, this may have led to a bias.

Furthermore, because secondary biomarker analysis requires splitting patients into 4 rather than 2 groups, the sample size becomes smaller. This effect was noted mostly in comparison with the Myriad MyChoice CDx. The concordance between the *BRCA*-like classifier and Myriad MyChoice was moderate, and associations with survival were the most relevant outcome measures for comparing the 2 tests. Our analysis of discordant cases revealed clinically relevant differences; however, the small sample size precluded conclusions.

These difficulties in comparing tests also hold for other tests included within and outside the ENGOT HRD Initiative. The European ENGOT HRD Initiative has allowed the validation of tests developed in the academic setting.^8,16,17,18^ The comparison between these DNA-based HRD tests is challenging when factors such as practical and logistical issues (eg, the required amount of DNA, turnaround times, access to algorithms, and equipment) are considered. Other tests range from mutations, genomic scar signatures, and mutational signatures to functional tests.^5^ All these tests have been shown to be associated with mechanistic explanations of HRD and responses to HRD-targeted therapy. Despite this, these biomarkers may represent a history of HRD-driven tumorigenesis rather than the actual HRD status, as it may change during tumor evolution.^21^ Functional assays could be used to assess the status of the HR pathway.^5,21,22^ As shown in our study, discordant results occur when comparing tests, and it has also been suggested that genomic scars and functional readouts may identify small nonoverlapping groups.^23^ In summary, these observations suggest that an optimal HRD test is not yet available and prompt further research to develop and validate tests to detect HRD with increasing accuracy.

## Conclusions

In this cohort study, the addition of olaparib was associated with longer PFS and OS in patients with advanced, high-grade epithelial OC with a *BRCA*-like tumor but not in patients with a non–*BRCA-*like tumor. Therefore, *BRCA*-like status may serve as a predictive biomarker in this patient population. The benefits of the *BRCA-*like classifier include a lower dropout rate and a larger group of patients with biomarker-positive tumors compared with the reference HRD test. This classifier will be further prospectively tested in the AGO-OVAR28 study (NCT05009082).^24^

## Notes

Supplement 1. **eMethods.** Supplementary Methods **eFigure 1.** Average Unsegmented and Segmented (B) Copy Number Profiles of 47 Samples Sequenced at the NKI and CCG Sites **eFigure 2.** Correlation Between the Distribution of 47 Samples Sequenced at the NKI and CCG **eFigure 3.** Distribution of (Segmented) Log Ratios of 47 Samples Sequenced at the NKI and CCG **eTable 1.** *BRCA1*-Like Classification of 47 Samples Sequenced at NKI and CCG **eFigure 4.** Copy Number Profiles of Discordant Samples Sequenced at the NKI and CCG **eFigure 5.** Density Plot of Sample Discordance Between NKI and CCG **eFigure 6.** Flow Diagram of Samples in the Study **eTable 2.** Programming Rules for Censoring PFS1 **eTable 3.** Cross Table of *BRCA*-like Status and Myriad MyChoice CDx **eTable 4.** Univariable Cox Regression Analysis of Samples With Discordant *BRCA*-Like and Myriad MyChoice CDx Results

Supplement 2.
Data Sharing Statement
