# Supplementary material for: Olaparib Addition to Maintenance Bevacizumab Therapy in Ovarian Carcinoma With BRCA-Like Genomic Aberrations
Source: JAMA Netw Open. 2024 Apr 9;7(4):e245552. doi: 10.1001/jamanetworkopen.2024.5552 (PMC11004830; doi:10.1001/jamanetworkopen.2024.5552)
Supplement: Supplement 1. — eMethods. Supplementary Methods eFigure 1. Average Unsegmented and Segmented (B) Copy Number Profiles of 47 Samples Sequenced at the NKI and CCG Sites eFigure 2. Correlation Between the Distribution of 47 Samples Sequenced at the NKI and CCG eFigure 3. Distribution of (Segmented) Log Ratios of 47 Samples Sequenced at the NKI and CCG eTable 1. BRCA1-Like Classification of 47 Samples Sequenced at NKI and CCG eFigure 4. Copy Number Profiles of Discordant Samples Sequenced at the NKI and CCG eFigure 5. Density Plot of Sample Discordance Between NKI and CCG eFigure 6. Flow Diagram of Samples in the Study eTable 2. Programming Rules for Censoring PFS1 eTable 3. Cross Table of BRCA-like Status and Myriad MyChoice CDx eTable 4. Univariable Cox Regression Analysis of Samples With Discordant BRCA-Like and Myriad MyChoice CDx Results [file jamanetwopen-e245552-s001.pdf]

Supplementary Online Content

Schouten PC, Schmidt S, Becker K, et al. Olaparib addition to maintenance bevacizumab therapy among patients with ovarian carcinoma with *BRCA*-like genomic aberrations. *JAMA Netw Open*. 2024;7(4):e245552. doi:10.1001/jamanetworkopen.2024.555

eMethods. Supplementary Methods

eFigure 1. Average Unsegmented and Segmented (B) Copy Number Profiles of 47 Samples Sequenced at the NKI and CCG Sites

eFigure 2. Correlation Between the Distribution of 47 Samples Sequenced at the NKI and CCG

eFigure 3. Distribution of (Segmented) Log Ratios of 47 Samples Sequenced at the NKI and CCG

eTable 1. *BRCA1*-Like Classification of 47 Samples Sequenced at NKI and CCG

eFigure 4. Copy Number Profiles of Discordant Samples Sequenced at the NKI and CCG

eFigure 5. Density Plot of Sample Discordance Between NKI and CCG

eFigure 6. Flow Diagram of Samples in the Study

eTable 2. Programming Rules for Censoring PFS1

eTable 3. Cross Table of *BRCA*-like Status and Myriad MyChoice CDx

eTable 4. Univariable Cox Regression Analysis of Samples With Discordant *BRCA*-Like and Myriad MyChoice CDx Results

This supplementary material has been provided by the authors to give readers additional information about their work.

© 2024 Schouten PC et al. *JAMA Network Open.*

eMethods. Supplementary methods

Low coverage sequencing methods

The 85 phase 2 samples were sequenced at the Netherlands Cancer Institute (NKI) as described before After validation of concordance between the NKI and Cologne Center for Genomics (CCG) sequencing facilities (eMaterials), the phase 3 samples were sequenced at CCG as follows. A total of 384 DNA samples (100 ng each) isolated from formalin fixed paraffin embedded (FFPE) tumors were provided by ARCAGY Research (8 Rue Lamennais, 75008 Paris, France). All DNA samples were treated with NEBNext FFPE DNA Repair Mix (M6630L; New England Biolabs GmbH, Frankfurt am Main, Germany) according to the manufacturer´s protocol. Mechanical shearing of DNA was performed using a Bioruptor NGS sonication system (UCD-600; Diagenode, 4102 Seraing (Ougrée), Belgium). DNA fragmentation was evaluated using a Tape Station 4200 (G2991BA, Agilent Technologies, Santa Clara, CA, USA) and (high-sensitivity) D1000 ScreenTapes (5067-5582, 5067-5584, Agilent). The TruSeq Nano DNA High Throughput Library Prep Kit (96 samples) was used (20015965, Illumina, San Diego, CA, USA) for library preparation. The library pool was quantified using the Peqlab KAPA Library Quantification Kit and the Applied Biosystems 7900HT Sequence Detection System and then sequenced on an Illumina NovaSeq 6000 sequencing instrument with a PE100 sequencing protocol (one SP flow cell).

Proof of concept: decentralized distribution

Previously, we used a HiSeq2500 sequencer with a 65-bp read length at the Netherlands Cancer Institute (NKI) (1). As a proof of concept of decentralized distribution and to investigate the robustness of the assay, we conducted sequencing of Phase 3 samples at the Cologne Center for Genomics (CCG). For higher throughput, we used an Illumina NovaSeq6000 sequencer. We sequenced 47 paired samples from the AGOTR1 study using the NovaSeq system (see Materials and Methods) and compared the classification results with those previously obtained using the HiSeq system (1). Readouts for successful validation comprised the similarity of the (average) profiles between both facilities/techniques and the concordance of classification.

We plotted the average log ratio and segmented log ratio copy number profiles of the samples sequenced at NKI and CCG. Visual inspection confirmed that the average profiles overlapped (Figure 1).

© 2024 Schouten PC et al. *JAMA Network Open.*

eFigure 1. Copy number aberration profiles of 47 samples sequenced at the NKI and CCG.

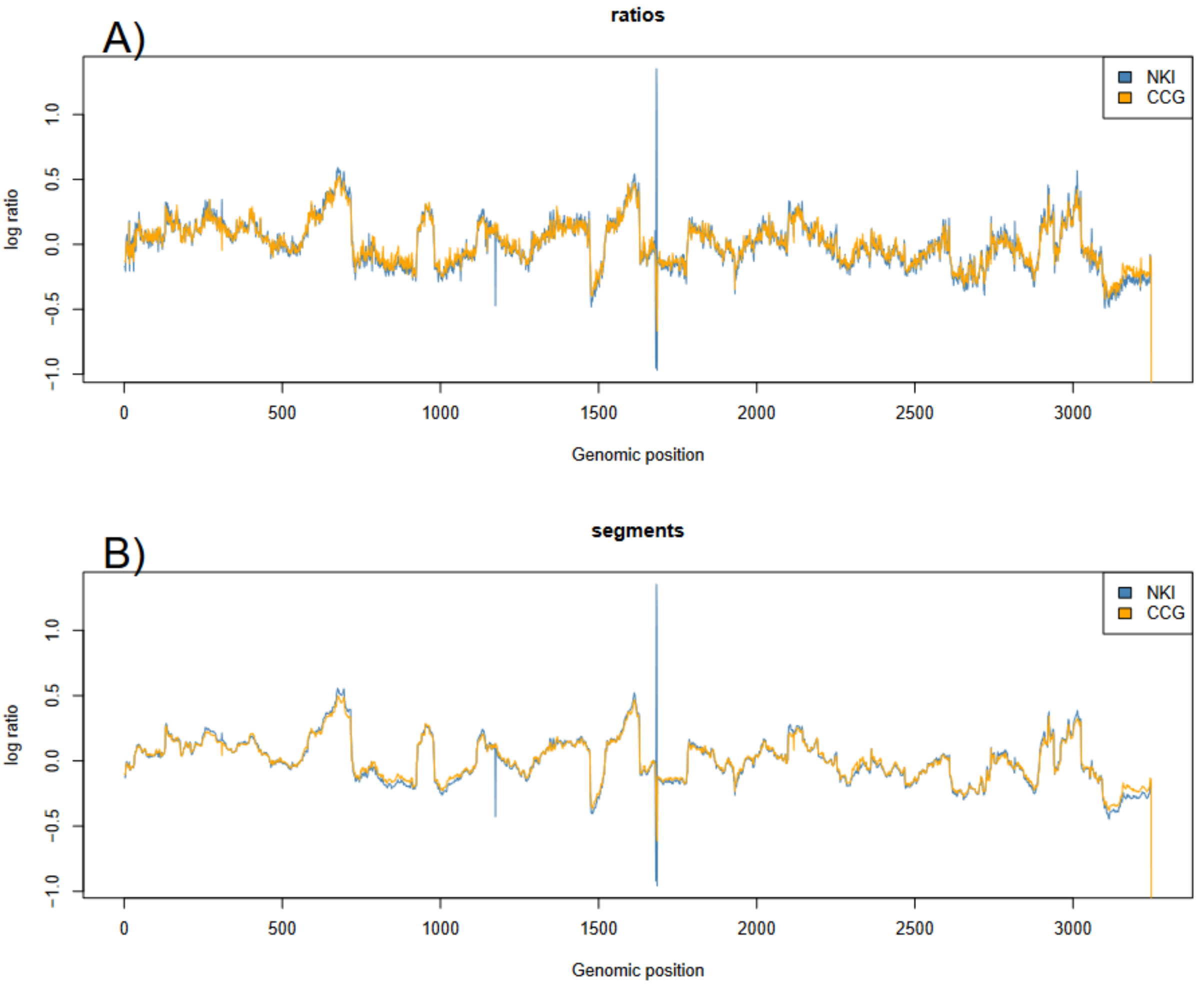

Average unsegmented (A) and segmented (B) copy number profiles of 47 samples sequenced at the NKI (blue) and CCG (orange) sites.

When we plotted the sorted and sorted segmented log ratios, there was a strong correlation between the two platforms, with the (segmented) log ratios mostly on the identity line of the plot (Figure 2). Notably, this measure resembles quantile (normalized) plots of the distribution and not the actual distribution or correlation between the profiles, that is, the range and centering of the (segmented) log ratios obtained by the two sequencing facilities.

© 2024 Schouten PC et al. *JAMA Network Open.*

eFigure 2. Correlation between the distribution of the 47 samples sequenced at the NKI and CCG.

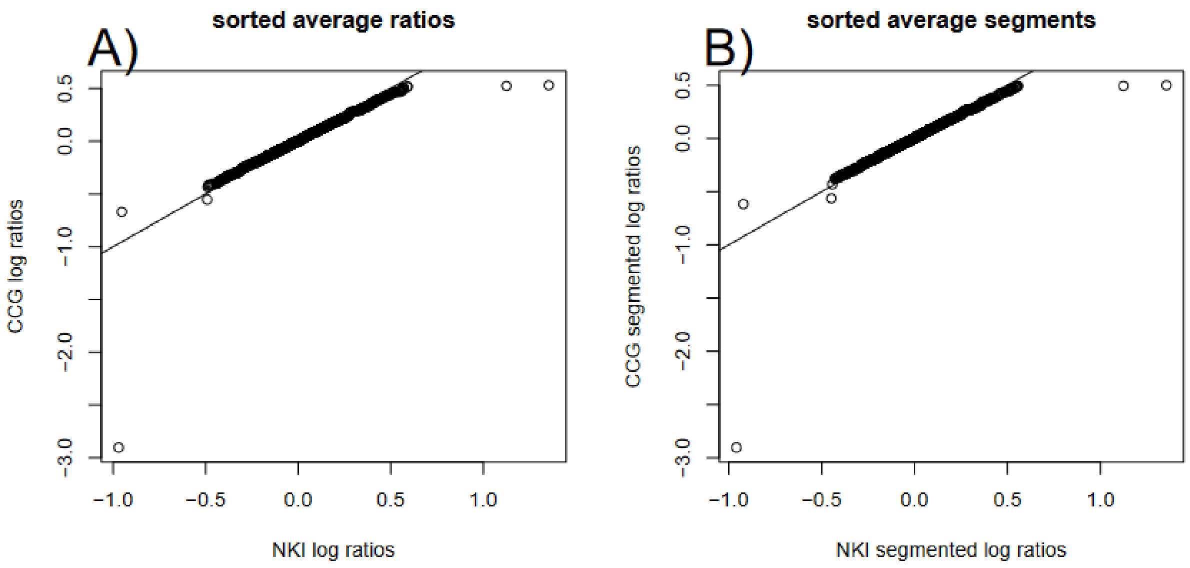

Sorted average log ratios (A) and segmented log ratios (B) of both centers. The diagonal line represents x= y.

We plotted the distributions of the log ratios and segmented log ratios (eFigure 3). The distributions are very similar.

© 2024 Schouten PC et al. *JAMA Network Open.*

eFigure 3. Distribution of (segmented) log ratios of 47 samples sequenced at the NKI and CCG.

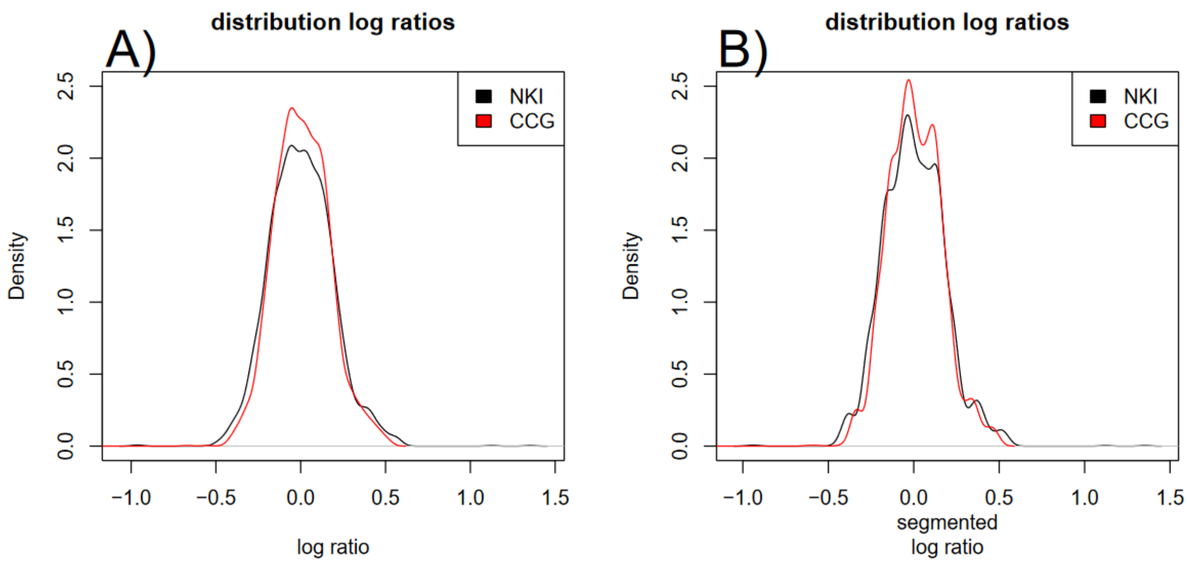

Given that the obtained copy number aberration profiles appeared to be very similar, specifically, the segmented ratios used for classification, we classified the 47 samples and cross-tabulated the results in eTable 1. 46/47 samples (98%) showed the same BRCA1-like classification, see eTable 1.

© 2024 Schouten PC et al. *JAMA Network Open.*

eTable 1. *BRCA1*-like classification of 47 samples sequenced at NKI and CCG

		CCG	
		Not *BRCA1*-like	*BRCA1*-like
NKI	Not *BRCA1*-like	23	1
	BRCA1-like	0	23

We plotted the copy number aberration profile (eFigure 4) of the discordant sample and observed both in the visual assessment and confirmed by the density plot (eFigure 5) that the CCG-sequenced profile had reduced amplitude. Although the overall validation was successful, there was residual experimental variation despite the profile passing quality control.

© 2024 Schouten PC et al. *JAMA Network Open.*

eFigure 4. Copy number profiles of discordant samples sequenced at the NKI and CCG.

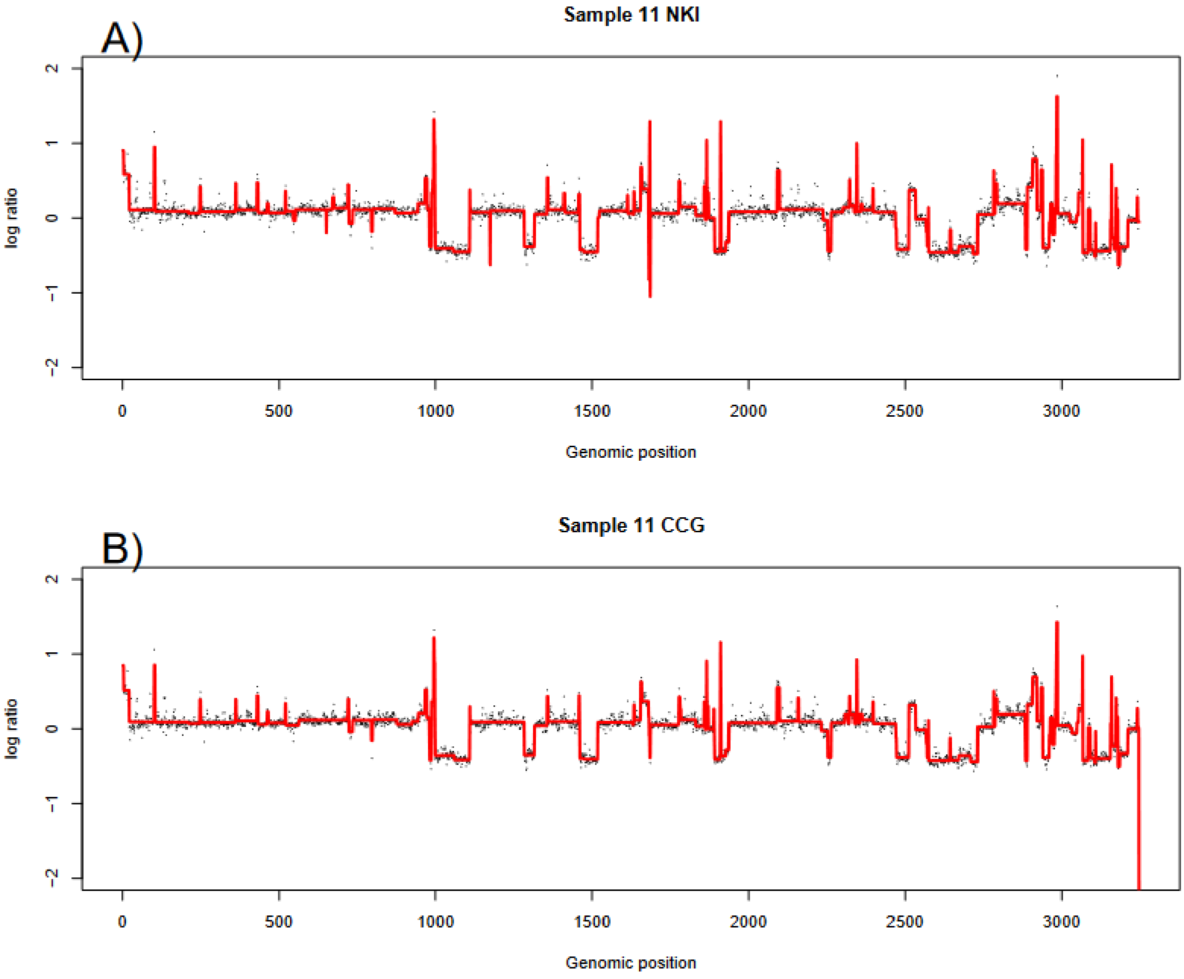

© 2024 Schouten PC et al. *JAMA Network Open.*

eFigure 5. Density plot of sample discordance between NKI and CCG.

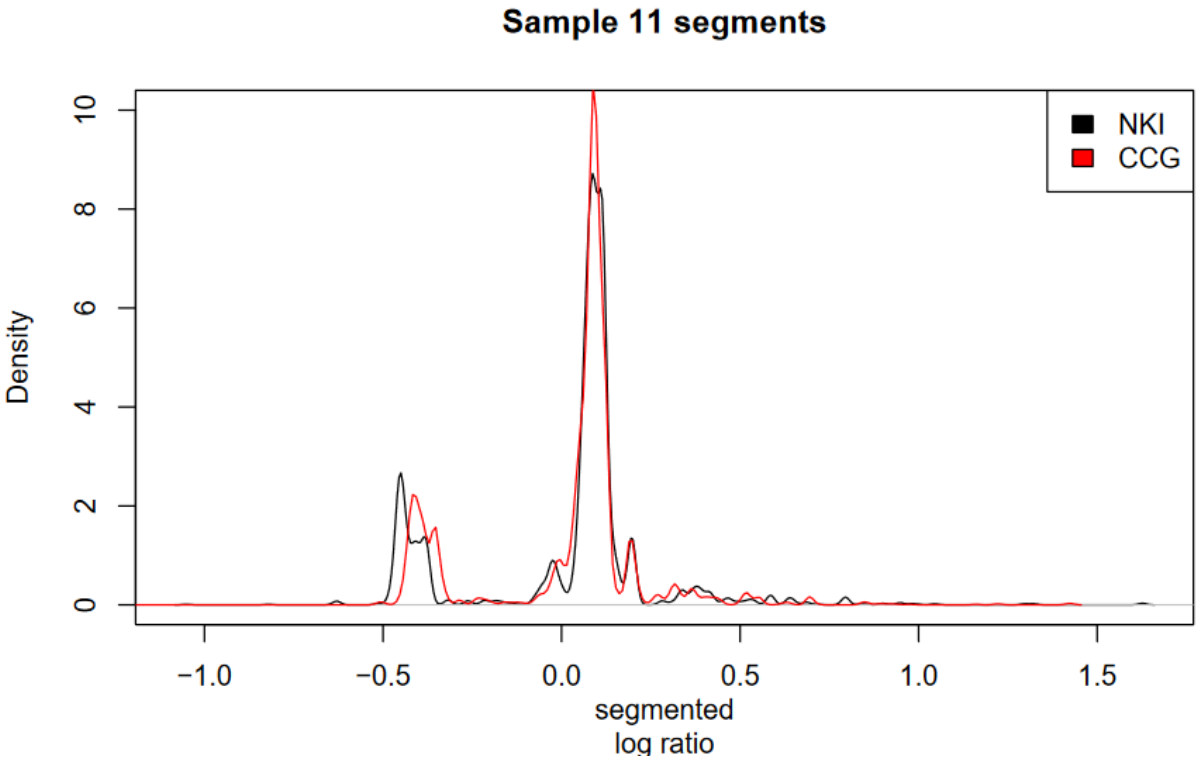

Reduced amplitude for the CCG sample is observed, e.g. at the peak around -0.5 and in the values > 0.25.

© 2024 Schouten PC et al. *JAMA Network Open.*

eFigure 6. Flow diagram of samples in the study.

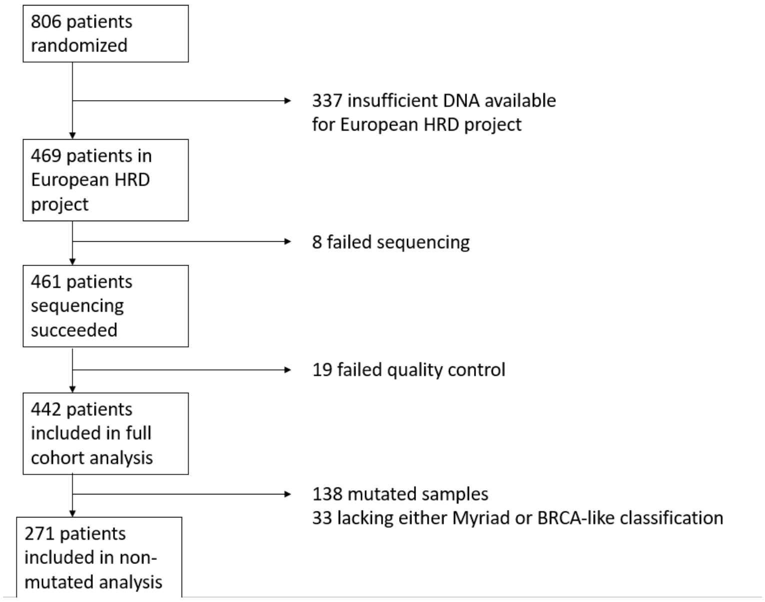

© 2024 Schouten PC et al. *JAMA Network Open.*

eTable 2. Programming rules for censoring PFS1.

PFS1 (investigator assessment based on RECIST version 1.1)

Situation	***Date of event or censoring***	***Outcome***
No RECIST baseline assessment or no post-baseline RECIST assessment	*Day 1 after randomisation if no death within two visits of baseline (49 weeks)*	*Censored*
	Date of death if death within two visits of baseline (49 weeks)	*Failed*
Documented progression based on investigator RECIST assessment	*Date of earliest RECIST assessment showing disease progression*	*Failed*
No documented progression based on investigator RECIST assessment	*Date of last RECIST assessment*	*Censored*
*Treatment discontinuation for undocumented progression**	*Date of last RECIST assessment*	*Censored*
*Treatment discontinuation for toxicity or other reason**	*Date of last RECIST assessment*	*Censored*
*New anticancer treatment started**	*Date of last RECIST assessment*	*Censored*
Death before first RECIST assessment	*Date of death*	*Failed*
*Death between adequate assessment visits**	*Date of death*	*Failed*
Death or progression after more than two missed visits	*Date of last RECIST assessment*	*Censored*

***without documented progression based on investigator RECIST assessment**

© 2024 Schouten PC et al. *JAMA Network Open.*

eTable 3. Cross table of BRCA-like status and Myriad MyChoice CDx

			Myriad MyChoice				
			neg	pos	neg	pos	
		BRCA Mutation	no	no	yes	yes	missing
BRCA1-like	no	no	107	14	0	0	14
	yes	no	68	82	0	0	19
	no	yes	0	0	0	8	1
	yes	yes	0	0	1	125	3
	missing		7	5	0	8	7

© 2024 Schouten PC et al. *JAMA Network Open.*

eTable 4. Univariable Cox regression analysis of samples with discordant BRCA-like and Myriad MyChoice CDx results.

		Concordant samples						Discordant samples				
		Median, (Q1, Q3)	Events/n (pm)	hazard ratio	95% CI	p		Median (Q1, Q3)	Events/n (pm)	hazard ratio	95% CI	p
Progression free survival	Non-*BRCA*-like/Myriad not HRD, placebo	18.8 (9.7, 37.5)	23/30 (724)	1			Non-*BRCA*-like/Myriad HRD, placebo	15.1 (8.0, 16.6)	5/5 (156)	1		
	Non-*BRCA*-like/Myriad not HRD, olaparib	17.3 (9.1, 17.3)	67/77 (1483)	1.49	0.90–2.48	0.12	Non-*BRCA*-like/Myriad HRD, olaparib	38.9 (16.7, not reached)	6/9 (538)	0.19	0.06–0.65	0.008
	BRCA-like/Myriad HRD, placebo	19.1 (11.8, 30.1)	27/31 (1745)	1			*BRCA*-like/Myriad not HRD, placebo	15.0 (9.7, 19.3)	20/21 (338)	1		
	BRCA-like/Myriad HRD, olaparib	38.7 (16.7, not reached)	28/51 (6042)	0.6	0.34–1.07	0.08	*BRCA*-like/Myriad not HRD, olaparib	16.5 (8.9, 23.9)	40/47 (967)	0.56	0.32–0.974	0.04

© 2024 Schouten PC et al. *JAMA Network Open.*

Overall survival	Non-*BRCA*-like/Myriad not HRD, placebo	54.2 (28.2, not reached)	15/30 (1368)	1			Non-*BRCA*-like/Myriad HRD, placebo	46.3 (39.5, not reached)	3/5 (370)	1		
	Non-*BRCA*-like/Myriad not HRD, olaparib	38.8 (19.7, 62.2)	56/77 (2932)	1.7	0.97–2.96	0.06	Non-*BRCA*-like/Myriad HRD, olaparib	54.1 (29.4, not reached)	5/9 (679)	0.4	0.11–1.46	0.17
	BRCA-like/Myriad HRD, placebo	55 (35.2, not reached)	17/31 (1745)	1			*BRCA*-like/Myriad not HRD, placebo	40.4 (25.7, 54.1)	17/21 (821)	1		
	BRCA-like/Myriad HRD, olaparib	not reached (36.8, not reached)	19/51 (7784)	0.61	0.32–1.18	0.14	*BRCA*-like/Myriad not HRD, olaparib	44.3 (20.5, 59.1)	33/47 (1882)	0.67	0.38–1.19	0.17

pm: person months.

© 2024 Schouten PC et al. *JAMA Network Open.*
