# Supplementary material for: Olaparib Addition to Maintenance Bevacizumab Therapy in Ovarian Carcinoma With BRCA-Like Genomic Aberrations
Source: JAMA Netw Open. 2024 Apr 9;7(4):e245552. doi: 10.1001/jamanetworkopen.2024.5552 (PMC11004830; doi:10.1001/jamanetworkopen.2024.5552)
Supplement: Supplement 2. — Data Sharing Statement [file jamanetwopen-e245552-s002.pdf]

Data Sharing Statement

Schouten. Olaparib Addition to Maintenance Bevacizumab Therapy in Ovarian Carcinoma With BRCA-Like Genomic Aberrations. *JAMA Netw Open*. Published April 09, 2024. doi:10.1001/jamanetworkopen.2024.5552

Data
Data available: Yes
Data types: Other (please specify)
Additional Information: Study data upon request to the respective study/data coordinators via corresponding author.
How to access data: Study data upon request to the respective study/data coordinators via corresponding author.
When available: With publication

Supporting Documents
Document types: None

Additional Information
Who can access the data: researchers whose proposed use of the data has been approved.
Types of analyses: For specified purposes.
Mechanisms of data availability: Will be assessed on a case-by-case basis, with a signed data access agreement.
